# Isokinetic eccentric exercise substantially improves mobility, muscle strength and size, but not postural sway metrics in older adults, with limited regression observed following a detraining period

**DOI:** 10.1007/s00421-020-04466-7

**Published:** 2020-08-09

**Authors:** Anthony David Kay, Anthony John Blazevich, Millie Fraser, Lucy Ashmore, Mathew William Hill

**Affiliations:** 1 Centre for Physical Activity and Life Sciences, Faculty of Art, Science and Technology, University of Northampton, Northamptonshire, UK; 2 Centre for Exercise and Sports Science Research (CESSR), School of Exercise and Health Sciences, Edith Cowan University, Joondalup, Australia; 3 Centre for Sport, Exercise and Life Sciences, School of Life Sciences, Coventry University, Warwickshire, UK

**Keywords:** Resistance training, Ageing, Functional decline, Posturography, Sarcopenia

## Abstract

**Introduction:**

Eccentric exercise can reverse age-related decreases in muscle strength and mass; however, no data exist describing its effects on postural sway. As the ankle may be more important for postural sway than hip and knee joints, and with older adults prone to periods of inactivity, the effects of two 6-week seated isokinetic eccentric exercise programmes, and an 8-week detraining period, were examined in 27 older adults (67.1 ± 6.0 years).

**Methods:**

Neuromuscular parameters were measured before and after training and detraining periods with subjects assigned to ECC (twice-weekly eccentric-only hip and knee extensor contractions) or ECC_PF_ (identical training with additional eccentric-only plantarflexor contractions) training programmes.

**Results:**

Significant (*P* < 0.05) increases in mobility (decreased timed-up-and-go time [− 7.7 to − 12.0%]), eccentric strength (39.4–58.8%) and vastus lateralis thickness (9.8–9.9%) occurred after both training programmes, with low-to-moderate weekly rate of perceived exertion (3.3–4.5/10) reported. No significant change in any postural sway metric occurred after either training programme. After 8 weeks of detraining, mobility (− 8.2 to − 11.3%), eccentric strength (30.5–50.4%) and vastus lateralis thickness (6.1–7.1%) remained significantly greater than baseline in both groups.

**Conclusion:**

Despite improvements in functional mobility, muscle strength and size, lower-limb eccentric training targeting hip, knee and ankle extensor muscle groups was not sufficient to influence static balance. Nonetheless, as the beneficial functional and structural adaptations were largely maintained through an 8-week detraining period, these findings have important implications for clinical exercise prescription as the exercise modality, low perceived training intensity, and adaptive profile are well suited to the needs of older adults.

## Introduction

Neuromuscular and musculoskeletal functional decline in ageing presents with comorbidities associated with falls including, but not limited to, a loss of muscle mass and strength (Hairi et al. 2010) and impaired balance and mobility (Delbaere et al. 2010). Whilst a range of exercise interventions have been demonstrated to reverse age-related reductions in physical function (Chou et al. 2012), limited cardiorespiratory capacity, locomotor ability, and fear of falling can reduce exercise capacity and tolerance (LaStayo et al. 2003). These issues can subsequently limit the ability to exercise at a sufficient intensity to prevent further losses in neuromuscular function, accelerating decline and contributing to the development of frailty (Cesari et al. 2014), increased levels of disability (Freedman et al. 2002), and poor quality of life (Chou et al. 2012). Consequently, improvements in muscle strength evoked by traditional progressive resistance training can be somewhat limited and, importantly, are often not transferred to improved balance or mobility performances (Orr et al. 2008). Therefore, developing resistance exercise strategies with sufficient stimulus to effectively and simultaneously counteract multiple impairments, whilst minimising the challenges older adults face with traditional forms of exercise, remains a high priority in ageing research.

Resistance training is widely used to improve muscle mass and strength in older adults within multi-component fall prevention programmes (Sherrington et al. 2019), with high-intensity resistance training also providing the necessary stimulus for neuromuscular adaptations leading to enhanced mobility (Hess and Woollacott 2005). Although reduced cardiorespiratory function can limit exercise tolerance and adherence to traditional forms of high-intensity exercise in older adults (LaStayo et al. 2003), the metabolic demand of eccentric exercise is ~ 25% that of concentric exercise at a comparable workload (LaStayo et al. 1999). Consequently, perceived effort during eccentric-only exercise is reduced (LaStayo et al. 2003) and exercise tolerance has been shown to be improved in older adults and individuals with cardiorespiratory impairments when compared with traditional resistance-based exercises that include cyclical concentric and eccentric contractions (Rooyackers et al. 2003). Furthermore, seated resistance exercise (e.g. performed on isokinetic machines) can be used in populations with poor mobility or fear of falling, whilst allowing for an adequate stimulus to be provided for lower-limb muscle strength improvement, as previously demonstrated in both young adult (Kay et al. 2018) and older (LaStayo et al. 2003) populations. Collectively, these findings suggest that seated eccentric-only exercise may be ideally suited to the specific physical challenges older adults face with traditional aerobic- and resistance-based exercises.

The superiority of adaptations to eccentric resistance training over isometric or concentric exercise has been demonstrated repeatedly in young adult populations with greater increases in muscle mass (Roig et al. 2009), strength (Hortobagyi et al. 1996), and changes in muscle architectural characteristics (i.e. fascicle length and pennation angle [Ema et al. 2016]). Interestingly, strength and mass also remained significantly improved after 6 weeks of detraining in well-trained young adults after eccentric, but not concentric, exercise (Coratella and Schena 2016). These findings are particularly relevant for older populations who often undergo periods of incapacitation; however no data exist describing the detraining effects after the cessation of eccentric exercise in older adults. Nonetheless, comparable improvements in functional mobility (i.e. timed-up-and-go test; TUG) have been reported after eccentric resistance training compared to traditional resistance training (Gault et al. 2012), with TUG times remaining significantly improved after 4 months of detraining following high-intensity (~ 80% maximal voluntary contraction [MVC]) but not low-intensity (~ 55% MVC) traditional strength training (Fatouros et al. 2005). Whilst chair-based exercise is often characterised as low intensity and lacking sufficient stimulus to elicit substantial adaptation, seated isokinetic and isotonic eccentric exercises have been reported to rapidly improve muscle strength and mass in young (Kay et al. 2018) and older (Reeves et al. 2009) adult populations. As muscle weakness is strongly related to fall risk (Benichou and Lord 2016), these findings indicate that eccentric exercise may be used to reverse several important comorbidities associated with fall risk, preventing the development of frailty, disability and institutionalisation, possibly with limited regression during periods of detraining, outcomes likely having important clinical implications for exercise prescription in older populations.

The association between lower-limb strength and balance is well established (Lord et al. 1991); nonetheless, reviews have reported that strength training often fails to improve static balance (i.e. postural sway [Orr et al. 2008]), possibly because the hip and knee extensors are targeted due to their relative importance for stair climbing and rising from a chair, but not the ankle musculature that influences postural sway assessments (Winter 1995). Therefore, as eccentric resistance training provides the greatest improvements in strength (Hortobagyi et al. 1996), and the strength of the ankle musculature is also important for balance (Winter 1995), eccentric resistance training programmes targeting the ankle musculature may provide simultaneous improvements in both strength and balance, two primary risk factors associated with falls in older adults (Hairi et al. 2010; Johansson et al. 2017). However, currently no data exist describing the effects of lower-limb isokinetic eccentric exercise on static balance (i.e. using quantitative posturography), an important predictor of future falls (Johansson et al. 2017), nor regression following the cessation of eccentric training in older adults, limiting our understanding of mid- to long-term potential benefits through periods of reduced activity more common in older adults. To overcome some of these issues, the aims of the present study were to examine the effects of two 6-week low-intensity, isokinetic eccentric lower-limb resistance training programmes targeting the hip and knee extensor muscle groups (ECC) or the hip, knee and ankle extensor muscle groups (ECC_PF_), and 8 weeks of subsequent detraining, on functional mobility, static balance, lower-limb eccentric muscle strength, and vastus lateralis muscle thickness and architecture in moderately active older adults. We hypothesised that both isokinetic eccentric resistance training programmes would significantly reduce TUG time and increase muscle strength, thickness and architecture, and that the positive effects of training would be largely retained at 8 weeks following the cessation of training. We also hypothesised that postural sway would be significantly reduced only in the ECC_PF_ group (i.e. with the inclusion of plantarflexor training).

## Materials and methods

### Subjects

Twenty-seven community-dwelling older adults volunteered for the study after completing a pre-test medical questionnaire and providing written informed consent. Subjects were matched for age, sex and mass, then assigned to either ECC (9 females, age = 67.4 ± 6.5 years, height = 1.59 ± 0.06 m, mass = 77.1 ± 15.5 kg; 3 males, age = 65.7 ± 6.7 years, height = 1.73 ± 0.07 m, mass = 91.3 ± 14.7 kg) or ECC_PF_ (11 females, age = 68.5 ± 6.0 years, height = 1.58 ± 0.06 m, mass = 70.1 ± 13.8 kg; 4 males, age = 72.3 ± 5.4 years, height = 1.75 ± 0.08 m, mass = 90.4 ± 7.0 kg) training groups. A PAR-Q revealed subjects were moderately active (~ 30 min moderate-intensity [e.g. walking] activity 3–5/week), were able to independently ambulate, and had no history of neurological (e.g. stroke, Parkinson’s disease), cognitive (e.g. dementia), cardiorespiratory (e.g. coronary heart disease, chronic obstructive pulmonary disease), or musculoskeletal (e.g. strain injury, tendonitis) conditions. Ethical approval was granted by the University of Northampton’s Ethics Committee with the study completed in accordance with the Declaration of Helsinki.

### Protocol

#### Overview

The subjects were initially familiarised with the testing and training protocols 1 week prior to data collection and then visited the laboratory four times under experimental conditions: twice before the training programmes commenced to determine baseline measures and test–retest reliability, and 1 week and 8 weeks after the programmes to determine training and detraining effects, respectively. During the experimental sessions, measures of postural sway were assessed using force platform analysis, dynamic balance (mobility) was assessed using the timed-up-and-go (TUG) test, vastus lateralis (VL) architecture (muscle thickness, pennation angle and fascicle length) were recorded using B-mode ultrasonography, and lower-limb eccentric force was measured using dynamometry. During the 6-week training programmes, subjects were advised to maintain their normal activities and diet to minimise the possible influence of external variables on the outcome measures. During training, subjects completed twice-weekly training sessions on a recumbent stepper (BTE Eccentron, Physiquipe, Manchester, UK). The ECC group completed an alternating unilateral eccentric leg press motion for a duration of 5 min (week 1) or 10 min (weeks 2–6), working at a rate of 40 contractions/min (described in detail below), and at an intensity of ~ 50% MVC. The ECC_PF_ group performed identical training with an additional 5 min of straight-legged unilateral eccentric plantarflexor contractions (described in detail below). During each training session, the difficulty (RPE), average force (% MVC), and accuracy (% of contractions on target) were recorded, with the average of the two sessions completed each week used to assess week-by-week outcomes.

### Measures

#### Muscle architecture

**Fig. 1:**
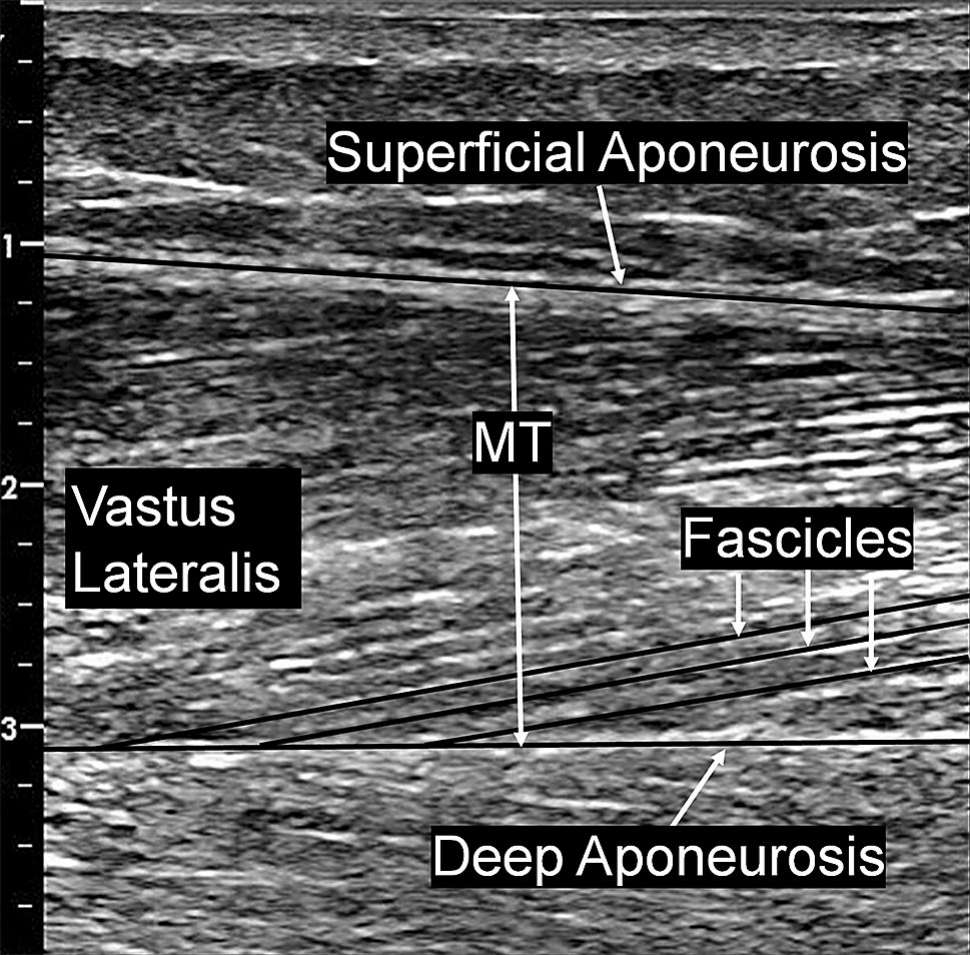
Ultrasound image of vastus lateralis muscle architecture. Muscle thickness (MT) was measured at the centre of the image between superficial and deep aponeuroses, with pennation angle (PA) calculated as the average from three fascicles, and standard trigonometry was used to calculate fascicle length

Ultrasound imaging (Vivid I, General Electric, Bedford, UK) recorded the vastus lateralis (VL) muscle using a wide-band linear probe (8L-RS, General Electric) with a 39 mm wide field of view and coupling gel (Dahlhausen, Cologne, Germany) between the probe and skin. Using previously established methods (Kay et al. 2018), during each experimental session the subjects were placed in a semi-reclined seated position with the hip at 70° and knee at 90°. The ultrasound probe was placed equidistant from the superior lateral border of the patella and the greater trochanter, and manipulated until the superficial and deep aponeuroses could be visualised to enable the longitudinal imaging of the VL. A still image of VL was then recorded, followed by the probe being removed from the skin and then repositioned before a second image was recorded to enable within-session reliability to be determined. Muscle thickness was measured at the centre of the image as the distance between superficial and deep aponeuroses, with pennation angle calculated as the average of three clearly visible fascicles inserting onto the deep aponeurosis (Fig. 1). Standard trigonometry was used to estimate fascicle length (FL), as the probe width did not enable the full fascicle length to be visualised. The average pennation angle (PA) from three fascicles and muscle thickness (MT) measurements were used to calculate fascicle length using the following equation: FL = MT / sin(PA).

#### Postural sway

Two minutes later, the subjects performed six 30-s quiet standing trials on a force platform (AccuGait, AMTI, Watertown, MA) under two alternating conditions (eyes open [EO], eyes closed [EC]) with 15 s rest between each trial. To ensure continuity between trials, subjects were unshod and instructed to stand quietly with the hands clasped together in front of the body and with the feet positioned in a V shape (abducted to ~ 30° at the hallux with the medial aspect of each calcaneus touching) over a pre-marked outline on the force platform (Hill et al. 2018). During eyes open trials, the subjects were instructed to gaze at a target on a wall 1.5 m away to minimise vestibular disturbance. Ground reaction force data were sampled at 100 Hz (Netforce, AMTI) and filtered using a fourth-order low-pass (6 Hz) Butterworth filter (BioAnalysis, V2.2, AMTI) prior to calculation of postural sway characteristics. The lowest postural sway metrics of the three trials in each condition was used to quantify postural sway during eyes open and eyes closed conditions. The total displacement of centre of pressure (COP) in the anteroposterior (COP_AP_ [cm]) and mediolateral (COP_ML_ [cm]) directions, COP velocity (cm·s^−1^), and 95% ellipse of the force trace (cm^2^) were calculated and used as measures of postural sway during static balance trials.

#### Functional mobility

Two minutes later, the subjects performed three TUG tests with 30 s of rest between trials to assess dynamic balance/functional mobility. Subjects were initially seated and asked to stand up without the use of their hands, walk as quickly (but safely) as possible to a marker 3 m away, turn, walk back to the chair, and sit down. A stopwatch was used to measure the time taken to complete the trial (to the nearest 0.01 s), with the fastest time (s) of the three trials used as a measure of functional mobility (Podsiadlo and Richardson 1991).

#### Eccentric lower-limb extensor strength

**Fig. 2:**
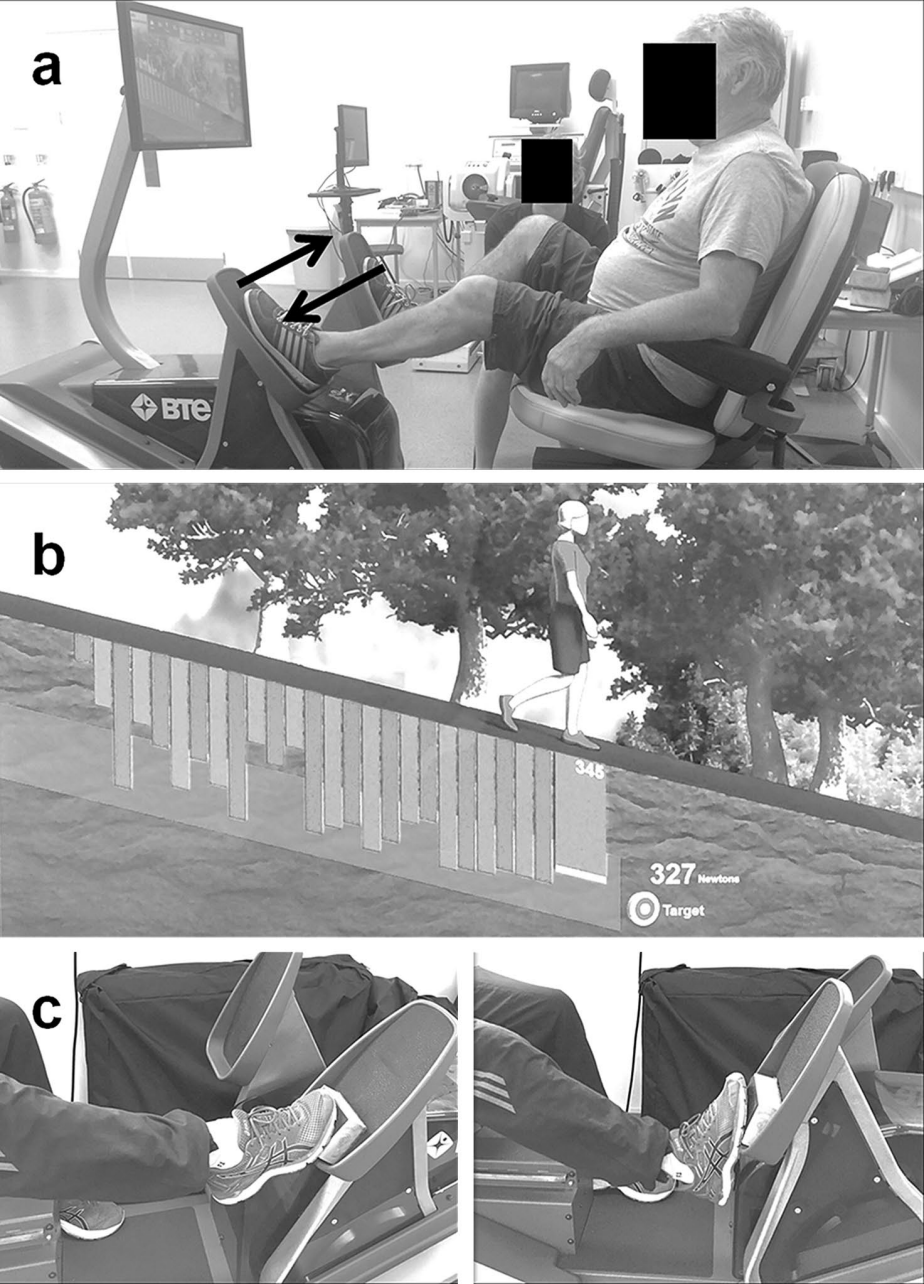
Subject positioning in the recumbent stepping machine during eccentric training. Subjects were seated in the machine with the chair adjusted for limb length to ensure the knee could not extend beyond ~ 30° or flex to more than ~ 90° during the exercise (**a**) to reduce the risk of knee joint injury. Real-time visual feedback of the subject’s force trace was imposed over a target force range (**b**) to enable subjects to match the required 50% maximal voluntary contraction (MVC) intensity during each contraction. To more effectively engage the ankle musculature, straight-legged plantarflexor contractions were included during the ECC_PF_ programme (**c**)

After a further 2 min rest, the subjects completed a lower-limb eccentric strength test on a recumbent stepping dynamometer (BTE Eccentron). This machine allows the subject to be seated during testing (and training) and is designed to imitate downhill stepping or walking (Fig. 2a). At the beginning of each experimental trial, injury risk to the knee was minimised by placing the subjects in a position that ensured the motion of the dynamometer resulting in the knee not extending beyond ~ 30° or flexing to more than ~ 90°. During the trial, the subjects initially performed a 3-min warm-up of alternating unilateral lower-limb eccentric contractions at 25% (1 min) and then 50% (2 min) MVC (determined during familiarisation) at a rate of 40 alternating steps/min (i.e. 20 per limb). Subjects then completed three maximal trials with each trial consisting of six alternating unilateral eccentric contractions on each limb; 60 s of rest was provided between trials. The peak eccentric force (N) from the three trials was used as a measure of lower-limb eccentric strength.

### Training programmes

#### ECC programme

The 6-week eccentric training programme was performed twice weekly in a seated position on the recumbent stepper, with the pedals moving in opposing directions and subjects instructed to resist the pedal moving towards them and relax as the pedal moves away. This motion, and resulting muscular contraction pattern, results in alternating unilateral eccentric-only contractions primarily of the lower-limb hip and knee extensor muscle groups. During the two training sessions completed in week 1, the subjects performed a 1-min warm-up at 25% MVC and 40 steps/min, followed by 5 min at 50% MVC and a 1-min warm-down at 25% MVC. The training duration at 50% MVC was increased to 10 min in weeks 2–6. As strength was expected to increase during the training programme, subjects were also retested in weeks 3 and 5 to allow load adjustment to maintain 50% MVC intensity. To assist the subjects to generate the required force, real-time visual feedback of the force trace and a target range (20% range was considered “on target”, i.e. 40–60% MVC) were provided (Fig. 2b). During the training programme, actual force production was recorded after each training session, and the average of the two sessions completed per week was calculated to determine actual weekly training intensity (weekly range = 46.0–47.4% MVC). During the training programme, RPE was also recorded after each training session using an 11-point scale (0–10), and the average of the two sessions completed per week was used to determine weekly RPE.

#### *ECC*_*PF*_* programme*

As knee flexion range of motion is greater than ankle dorsiflexion during the leg press motion performed in ECC, the gastrocnemii were not exposed to eccentric loading. Thus, to more effectively engage the plantarflexor muscle group, the 6-week ECC_PF_ eccentric training programme was performed identically to ECC, but with an additional 5 min of unilateral straight-legged eccentric plantarflexor contractions performed at 40 steps/min for 5 min at 50% of the training load used during the leg press exercise (Fig. 2c). Subjects were retested for leg press strength in weeks 3 and 5 to allow load adjustment to maintain 50% MVC intensity for both leg press and straight-legged plantarflexor exercises. During the training programme, actual plantarflexor force production was recorded after each training session, the average of the two sessions completed per week was calculated to determine actual weekly training intensity (weekly range = 50.8–55.5% of leg press training load).

### Data analysis

All data were analysed using SPSS statistical software (version 22; IBM, Chicago, IL) and reported as mean ± SD. The normality of distribution for all data sets was assessed using Shapiro–Wilk tests with Levene’s test used to determine equal variances, and log transformations (log_10_) were performed where normal distribution was violated. Paired *t* tests (two-tailed) were performed on data sets from the two pre-training trials to determine the test–retest reliability of each measure (data collapsed across groups). A separate two-way mixed model analysis of variance (ANOVA) was used to test for the within-subject effects of time (× 3) and between-subject effects of group (× 2) in (i) functional mobility (TUG), (ii) postural sway metrics, (iii) lower-limb eccentric strength, (iv) VL muscle thickness, (v) VL pennation angle, and (vi) VL fascicle length. Separate two-way mixed model ANOVAs were used to test for the within-subject effects of time (× 6) and between-subject effects of group (× 2) in (i) leg press step accuracy and (ii) RPE during the 6-week training programmes. A repeated measures ANOVA was used to determine the within-subject effects of time (× 6) in straight-legged plantarflexor accuracy during the ECC_PF_ programme. Where significant differences were detected, post hoc *t* tests were used to determine the location of any differences. Partial eta squared (*η*_*p*_^2^*)* and Cohen’s d (*d*) were used to calculate effect sizes for ANOVA and *t* tests, respectively. Pearson’s product moment correlation coefficients (*r*) were computed to quantify the relationship between the absolute change score data in all variables. Statistical significance for all tests was accepted at *P* < 0.05.

### Reliability

#### Within-session reliability

To determine within-session reliability, intraclass correlation coefficients (ICC) and coefficients of variation (CV) were calculated for TUG time and postural sway metrics in the first pre-training experimental session between the second and third trials (data collapsed across ECC and ECC_PF_ groups). No significant difference was detected between trials in TUG time (3.1%) with high ICC (1.00) and low CV (3.0%) demonstrating excellent within-session reliability. No significant differences were detected in any measure of postural sway (COP_AP_, COP_ML_, COP velocity, 95% ellipse) in EO (0.3–2.4%) or EC (1.2–5.8%) conditions with high ICCs (EO = 0.62–0.97; EC = 0.70–0.97) and low-to-moderate CVs (EO = 5.5–20.6%; (EC = 6.3–18.1%), demonstrating good within-session reliability. No significant difference was detected in strength (5.3%) with high ICC (0.99) and low CVs (4.9%) demonstrating excellent within-session reliability. To determine within-session intratester measurement error, VL architectural characteristics (FL, PA and MT) were measured from two separate images recorded during the first pre-training experimental session. No significant difference was detected (0.4–1.2%) with high ICCs (0.97–1.00) and low CVs (1.3–2.9%), demonstrating excellent reliability.

#### Between-session reliability

To determine between-session test–retest reliability, two experimental sessions were conducted 1 week apart before the training programmes commenced (data collapsed across ECC and ECC_PF_ groups). During the mobility trials, despite high ICC (0.96) and low CV (4.0%) calculated for TUG times between weekly trials indicating excellent reliability, a significant reduction in time (*P* < 0.05) was detected (− 3.7% [− 0.33 s]), suggesting that more than one familiarisation session may be required to minimise possible learning effects. During the static balance trials, no significant differences were detected in any measure of postural sway (COP_AP_, COP_ML_, COP velocity, and 95% ellipse) in EO (2.7–8.5%) or EC (4.7–7.8%) conditions with high ICCs (EO = 0.85–0.91; EC = 0.75–0.97) and low-to-moderate CVs (EO = 5.9–20.5% EC = 9.9–17.7%), demonstrating good reliability. No significant difference was detected in eccentric strength, FL, PA or MT (0.5–3.7%) with high ICCs (0.89–0.99) and low CVs (3.0–5.6%), demonstrating excellent reliability.

### Sample size

Effect sizes (Cohen’s *d*) were calculated from previous studies employing similar interventions from mean changes in strength (Kay et al. 2016), hypertrophy (Cadore et al. 2014), and mobility (LaStayo et al. 2003). To ensure adequate statistical power for all analyses, power analysis was conducted for hypertrophy (i.e. the variable with the smallest effect size) using the following parameters (power = 0.80, alpha = 0.05, effect size = 1.58). The analysis revealed the total sample size required for statistical power was 16; however, 30 subjects were recruited to account for possible attrition. Three subjects withdrew due to unrelated personal reasons, with data analysis conducted on complete data sets for the remaining 27 subjects (ECC = 12, ECC_PF_ = 15).

## Results

The two-way mixed model ANOVA revealed a significant main effect of time (*F* = 40.67, *P* < 0.001, *η*_*p*_^2^ = 0.63), but not group (*F* = 3.19, *P* = 0.09, *η*_*p*_^2^ = 0.12) for mobility (i.e. the time to complete the TUG test). Post hoc within-subject analyses revealed significant (*P* < 0.01) reductions in the time to complete the TUG test after training in ECC (− 12.0 ± 8.9% [− 0.94 ± 0.82 s], *d* = 1.15) and ECC_PF_ (− 7.7 ± 6.4% [0.64 ± 0.56 s], *d* = 1.14) (Table 1). No significant regression was detected after 8 weeks of detraining (~ 100% of the mean improvement being retained), with TUG time remaining significantly (*P* < 0.001) faster than at pre-training in ECC (− 11.3 ± 7.6% [− 0.84 ± 0.63 s], *d* = 1.34) and ECC_PF_ (− 8.2 ± 6.1% [0.66 ± 0.53 s], *d* = 1.24).

**Table 1:** Mean (± SD) mobility and postural sway metrics measured in dynamic and static balance tests during the pre-training, post-training, and detraining assessments

Measurement	ECC training programme			ECC_PF_ training programme		
	Pre-training	Post-training	Detraining	Pre-training	Post-training	Detraining
TUG (s)	7.28 ± 1.41	6.34 ± 1.08*	6.26 ± 1.09*	7.91 ± 1.07	7.27 ± 0.90*	7.24 ± 0.95*
EO ML sway (cm)	1.99 ± 0.74	1.96 ± 0.46	2.00 ± 0.66	2.08 ± 0.65	2.22 ± 0.73	2.00 ± 0.69
EC ML sway (cm)	2.26 ± 0.93	2.46 ± 0.70	2.43 ± 0.98	2.57 ± 0.98	2.74 ± 1.30	2.68 ± 1.14
EO AP sway (cm)	2.31 ± 0.47	2.37 ± 0.49	2.43 ± 0.57	2.37 ± 0.82	2.47 ± 0.48	2.30 ± 0.71
EC AP sway (cm)	3.11 ± 0.75	3.28 ± 0.56	3.04 ± 0.76	2.84 ± 0.73	3.01 ± 0.88	3.19 ± 0.95
EO ellipse (cm^2^)	3.44 ± 1.84	3.38 ± 1.39	3.69 ± 1.84	3.96 ± 2.61	4.38 ± 2.63	3.76 ± 2.34
EC ellipse (cm^2^)	5.49 ± 3.13	5.52 ± 2.15	5.21 ± 2.79	5.70 ± 3.91	6.14 ± 4.27	6.08 ± 4.38
EO velocity (cm·s^−1^)	1.72 ± 0.56	1.78 ± 0.47	1.65 ± 0.47	1.61 ± 0.55	1.70 ± 0.50	1.63 ± 0.58
EC velocity (cm·s^−1^)	2.38 ± 1.06	2.43 ± 0.81	2.23 ± 0.89	2.17 ± 0.85	2.31 ± 0.77	2.31 ± 0.88

*SD* standard deviation, *ECC* eccentric leg press training programme, ECC_PF_ eccentric leg press and plantarflexor training programme, *TUG* timed-up-and-go, * = *P* < 0.05 (versus pre-training), *EO* eyes open, *EC* eyes closed, *ML* mediolateral, *AP* anteroposterior

No significant main effects of time (*F* = 1.11–3.54, *P* > 0.05, *η*_*p*_^2^ = 0.06–0.13) or group (*F* = 0.005–0.46, *P* > 0.05, *η*_*p*_^2^ = 0.001–0.020) were detected in any postural sway metric (COP_ML_, COP_AP_, 95% ellipse, COP velocity) in either EO or EC conditions (Table 1).

**Fig. 3:**
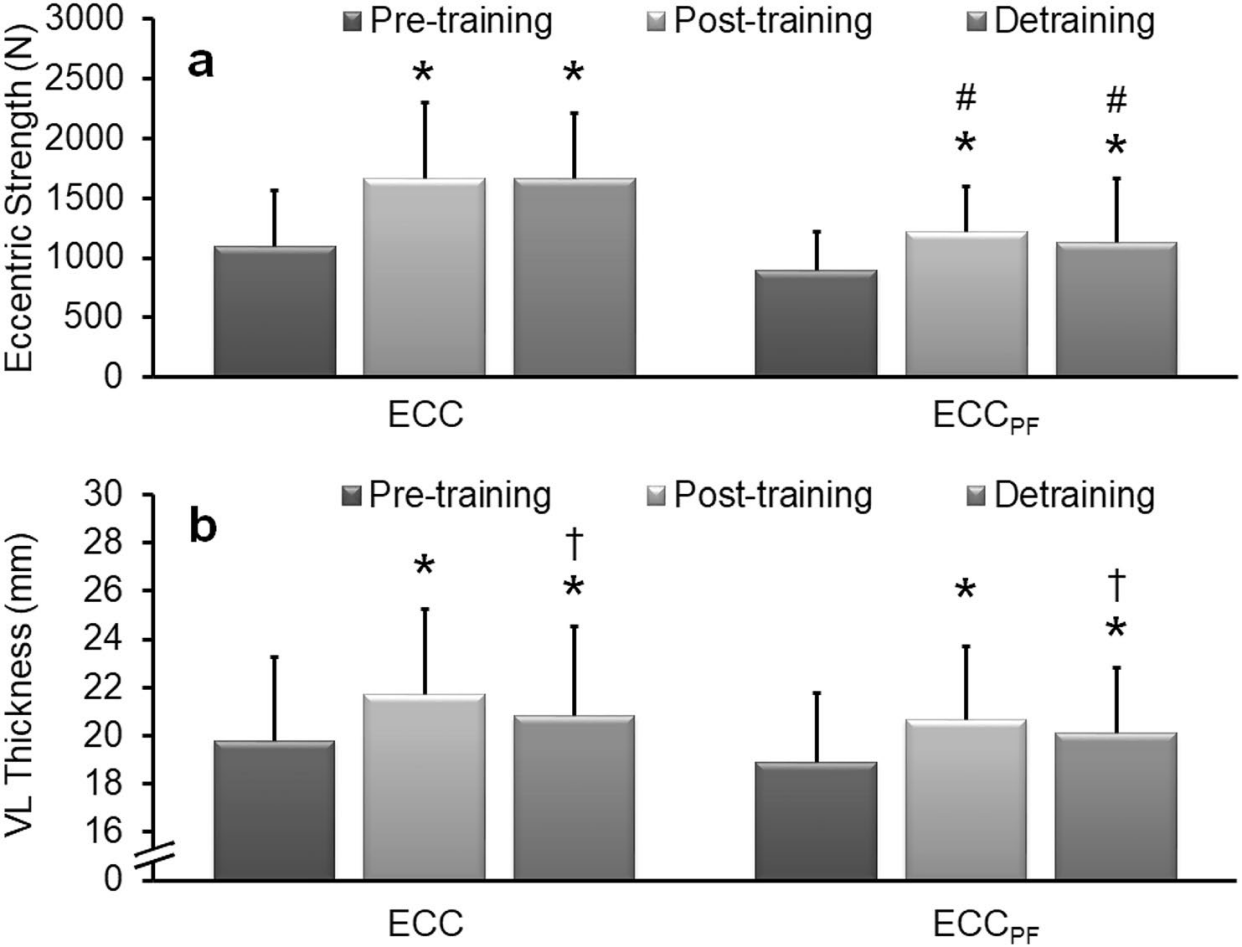
Mean (± SD) eccentric leg press strength and vastus lateralis muscle thickness measured during pre-, post-, and 8 weeks post-training assessments. Significant increases in peak eccentric strength (**a**) and muscle thickness (**b**) were detected after the 6-week ECC and ECC_PF_ training programmes. After 8 weeks of detraining, strength and muscle thickness remained significantly greater than at pre-training. *Significantly greater than pre-training, ^†^significantly smaller than post-training, ^#^significantly different to ECC at the same time point. Significance accepted at *P* < 0.05

Significant main effects of time (*F* = 60.44, *P* < 0.001, *η*_*p*_^2^ = 0.72) and group (*F* = 7.20, *P* = 0.04, *η*_*p*_^2^ = 0.23) were detected in eccentric leg press strength. Post hoc within-subject analyses revealed a significant (*P* < 0.01) increase in strength after training in ECC (58.8 ± 39.9% [573 ± 319 N], *d* = 1.80) and ECC_PF_ (39.4 ± 25.0% [323 ± 158 N], *d* = 2.04) (Fig. 3a). No significant regression occurred after detraining (~ 71–85% of the mean increase being retained), with strength remaining significantly (*P* < 0.01) greater than at pre-training in ECC (50.4 ± 38.0% [513 ± 326 N], *d* = 1.57) and ECC_PF_ (30.5 ± 28.7% [227 ± 166 N], *d* = 1.36). Post hoc between-subject analyses revealed no significant difference between groups at baseline, but significant (*P* < 0.05) differences post-training between ECC (1667 ± 631 N) and ECC_PF_ (1223 ± 379 N) and after detraining between ECC (1666 ± 540 N) and ECC_PF_ (1127 ± 311 N) were observed.

A significant main effect of time (*F* = 51.20, *P* < 0.001, *η*_*p*_^2^ = 0.68), but not group (*F* = 0.33, *P* = 0.57, *η*_*p*_^2^ = 0.01) was detected in VL muscle thickness. Post hoc within-subject analyses revealed significant (*P* < 0.01) increases in VL thickness after training in ECC (9.8 ± 1.3% [1.89 ± 0.74 mm]; *d* = 2.54) and ECC_PF_ (9.9 ± 1.8% [1.80 ± 1.05 mm], *d* = 1.71) (Fig. 3b). Although a significant (*P* < 0.05) reduction in VL muscle thickness was detected after detraining in both groups, VL thickness remained significantly (*P* < 0.01) greater than at pre-training in ECC (6.1 ± 1.6%; *d* = 1.14) and ECC_PF_ (7.1 ± 1.7%; *d* = 1.30), with ~ 62–71% of the mean increase in muscle thickness being retained.

**Fig. 4:**
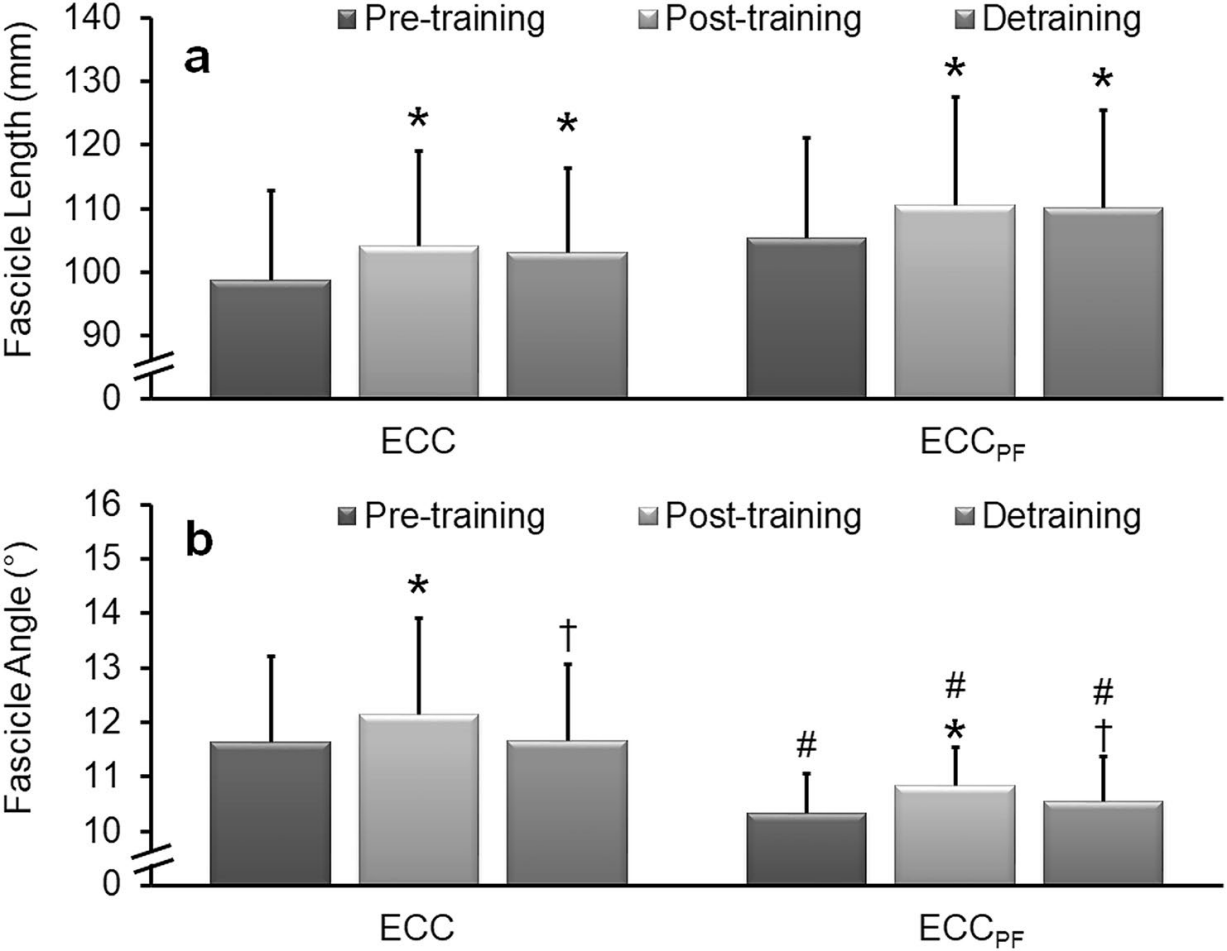
Mean (± SD) vastus lateralis fascicle length and pennation angle during pre-, post-, and 8 weeks post-training assessments. Significant increases in fascicle length (**a**) and pennation angle (**b**) were detected after the 6-week ECC and ECC_PF_ training programmes. After 8 weeks of detraining, no significant reduction in fascicle length occurred, which remained significantly longer than pre-training, indicating limited regression, whereas pennation angle reverted to baseline. *Significantly greater than pre-training, ^†^significantly less than post-training, ^#^significantly different to ECC at the same time point. Significance accepted at *P* < 0.05

A significant main effect of time (*F* = 9.49, *P* < 0.01, *η*_*p*_^2^ = 0.28), but not group (*F* = 1.61, *P* = 0.22, *η*_*p*_^2^ = 0.06) was detected in VL fascicle length. Post hoc within-subject analyses revealed significant (*P* < 0.01) increases in VL fascicle length after training in ECC (5.4 ± 4.3% [5.3 ± 4.5 mm]; *d* = 1.17) and ECC_PF_ (4.8 ± 4.4% [5.1 ± 4.7 mm], *d* = 1.07) (Fig. 4a). No significant regression occurred after detraining (~ 94–99% of the mean increase retained), with fascicle length remaining significantly (*P* < 0.01) longer than at pre-training in both ECC (5.8 ± 7.5% [5.2 ± 7.1 mm], *d* = 0.74) and ECC_PF_ (4.9 ± 6.8% [4.8 ± 7.3], *d* = 0.65).

A significant main effect of time (*F* = 8.25, *P* < 0.01, *η*_*p*_^2^ = 0.26) and group (*F* = 7.87, *P* < 0.05, *η*_*p*_^2^ = 0.25) was detected in VL pennation angle. Post hoc within-subject analyses revealed a significant (*P* < 0.01) increase in VL pennation angle after training in ECC (4.4 ± 5.7% [0.52 ± 0.69°], *d* = 0.75) and ECC_PF_ (5.0 ± 5.0% [0.50 ± 0.48°], *d* = 1.05) (Fig. 4b). Significant reductions in VL pennation angle were detected after detraining, with no significant difference detected compared to pre-training in ECC (0.5 ± 5.1% [0.01 ± 0.64°], *d* = 0.02) and ECC_PF_ (2.5 ± 8.3% [0.23 ± 0.83°], *d* = 0.28), indicating that pennation angle had largely returned to baseline. Post hoc between-subject analyses revealed significant (*P* < 0.05) differences in VL pennation angle (1.1–1.3°) between groups at pre-training (ECC = 11.6 ± 1.6°; ECC_PF_ = 10.3 ± 0.7°), post-training (ECC = 12.1 ± 1.8°; ECC_PF_ = 10.8 ± 0.7°), and after detraining (ECC = 11.7 ± 1.4°; ECC_PF_ = 10.6 ± 0.8°).

When the relationships between absolute change data were examined, significant correlations were observed between changes in VL pennation angle and both muscle thickness (*r* = 0.54; *P* < 0.01) and fascicle length (*r* = − 0.47; *P* < 0.05), but not between changes in muscle thickness and fascicle length (*r* = 0.35; *P* = 0.07). No significant correlations were observed between the changes in strength or TUG and any other measure (*r* = − 0.19 to 0.22; *P* > 0.05).

**Fig. 5:**
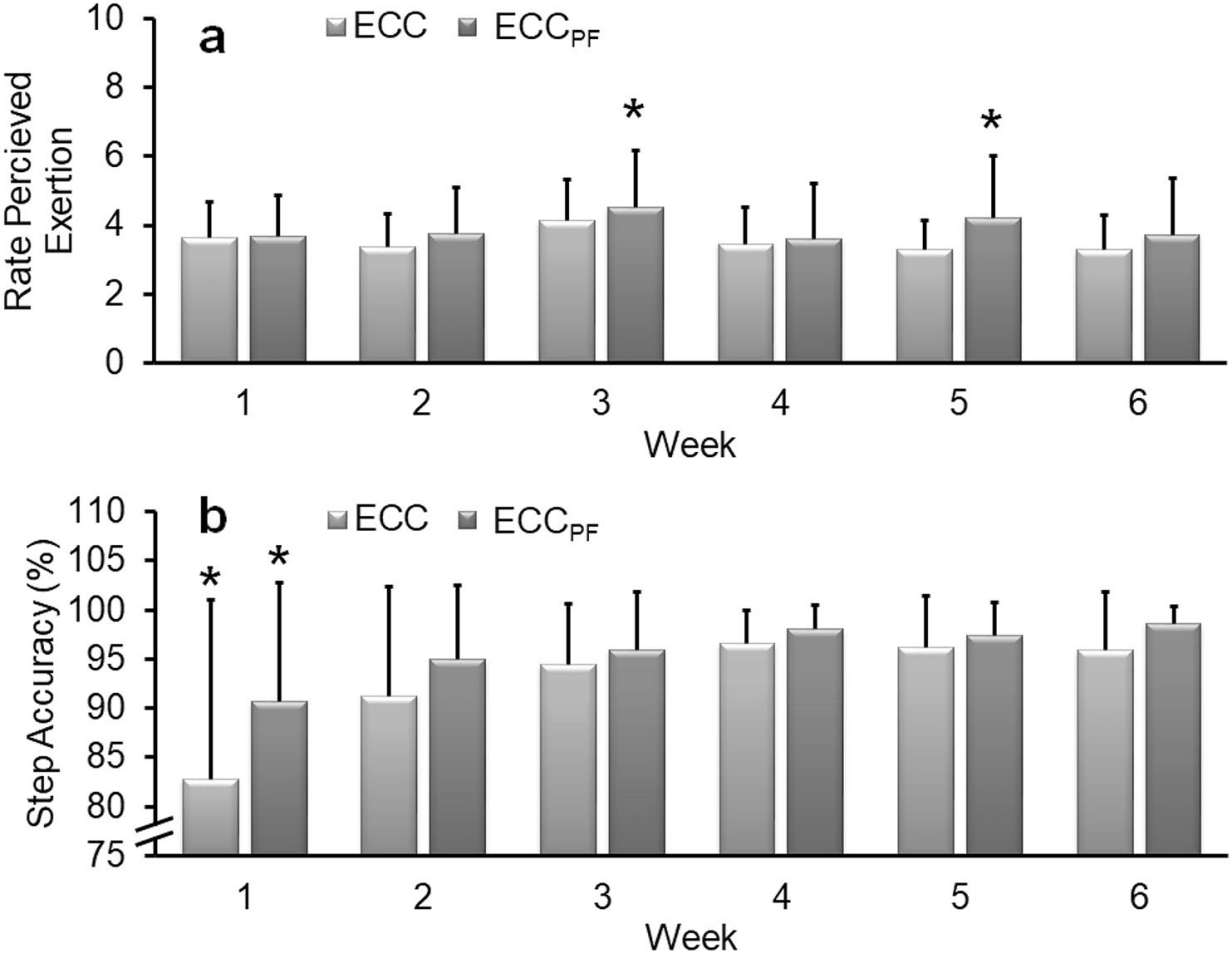
Mean (± SD) weekly rates of perceived exertion (RPE) and step accuracy during the 6-week training programmes. Although RPE remained consistently low to moderate during the programmes, a significant increase in weekly RPE was detected in week 3 and week 5 during the ECC_PF_ training programme (**a**). A significant increase in step accuracy was detected after week 1 (**b**) during both training programmes with accuracy greater in weeks 2–6 than week 1. No further change occurred during the training programmes. *Significantly different from other weeks. Significance accepted at *P* < 0.05

A significant main effect of time (*F* = 4.78, *P* < 0.01, *η*_*p*_^2^ = 0.16), but not group (*F* = 0.76, *P* = 0.39, *η*_*p*_^2^ = 0.03) was detected in weekly RPE. Post hoc within-subject analyses revealed significantly greater RPE (*P* < 0.05) in weeks 3 and 5 (compared to other weeks) in the ECC_PF_ group (i.e. weeks where absolute intensity was increased). No further difference in RPE was observed between any other week in either programme, with RPE remaining consistently low throughout the ECC (3.3–4.1 out of 10; i.e. ‘moderate’ to ‘somewhat hard’) and ECC_PF_ (3.6–4.5 out of 10) programmes (Fig. 5a).

A significant main effect of time (*F* = 12.43, *P* < 0.001, *η*_*p*_^2^ = 0.33), but not group (*F* = 1.79, *P* = 0.19, *η*_*p*_^2^ = 0.07) was detected in weekly leg press step accuracy. Post hoc within-subject analyses revealed significantly (*P* < 0.05) greater leg press step accuracy (i.e. the ability to match the required 50% MVC target force) in weeks 2–6 (ECC = 91.3–96.6%, ECC_PF_ = 95.0–98.7%) than week 1 (ECC = 82.8%, ECC_PF_ = 90.7%). No further difference in leg press accuracy was observed from weeks 2 to 6 in either programme (Fig. 5b). Similarly, a significant (*F* = 14.93, *P* < 0.001, *η*_*p*_^2^ = 0.52) increase in straight-legged plantarflexor step accuracy was detected during the ECC_PF_ training programme with accuracy greater in weeks 2–6 (91.7–96.8%) than week 1 (88.1%). No further difference in straight-legged plantarflexor accuracy was observed from weeks 2 to 6.

## Discussion

Impaired mobility is an important risk factor for falls, with individuals recording TUG times of > 13.5 s having a 90% probability of being a faller (Shumway-Cook et al. 2000). However, subjects in the present study were moderately active and completed the TUG in 7.3–7.8 s (mean range for both groups) during pre-training assessments, times at the faster end of the normative spectrum (7.1–9.0 s) for community dwelling-older adults aged 60–69 years (Bohannon 2006). Nonetheless, in agreement with our hypothesis, a 9–12% (− 0.64 s to − 0.94 s [*d* = 1.14–1.15]) improvement in mobility performance (faster TUG time) was observed after both eccentric training programmes, placing the subjects well above the expected range within this demographic. While one meta-analysis revealed that traditional forms of resistance training did not improve mobility in older adults (Chou et al. 2012), the improvement in mobility in the present study is consistent with previous studies examining the effects of eccentric resistance training in older adults (LaStayo et al. 2003; Gault et al. 2012]. Novel to the present study, however, is the assessment of detraining effects following the cessation of the interventions. Importantly, no regression in mobility occurred after the 8-week detraining period, indicating that a relevantly short period of eccentric training was sufficient to improve mobility in an already-mobile cohort with the retention of improvements maintained through a detraining period. As older adults can be more prone to training interruptions (due to injury and/or illness, caring for a spouse or child, etc.), the retention of mobility is valuable to practitioners, care providers and exercise professionals involved with exercise programming and implementation. From a fall-risk perspective, it may be a worthwhile strategy to prescribe eccentric resistance training to older adults who are expecting a future period of inactivity (i.e. prehabilitation to assist in the preservation of mobility performance).

Previous studies examining the effects of eccentric resistance training in older adults (Gault et al. 2012; LaStayo et al. 2003) have not examined quiet standing postural sway, which is an important risk factor associated with falls (Johansson et al. 2017). Despite improvements in dynamic balance (i.e. mobility) being evoked in the present study, and the inclusion of plantarflexor training in the ECC_PF_ group, no significant change in any COP measure of postural sway (6.9–14.0%; *η*_*p*_^2^ = 0.06–0.13) was observed in the present study. This result runs counter to our hypothesis. Nonetheless, these data are consistent with previous systematic reviews (Orr et al. 2008) and meta-analyses (Low et al. 2017) examining the impact of traditional resistance exercises on static balance performance. The underlying mechanisms governing the significant improvements in dynamic balance (i.e. TUG time), but not static balance (i.e. postural sway), in the present study are not clear, although distinct neuromuscular qualities may need to be targeted separately for their respective improvements (Muehlbauer et al. 2015). Importantly, eccentric training is characterised by the preferential recruitment of fast twitch motor units (Douglas et al. 2017), which would logically improve performance during fast velocity dynamic tasks (i.e. TUG [Miller et al. 2015]), but not during quiet standing as the antigravity muscles that minimise body sway show a greater predominance of type I muscle fibres (Paillard 2017). Alternatively, relatively low-intensity and short duration training programmes may not be sufficient to generate adaptations in standing balance, and the seated exercise did not involve body movements that stress, and therefore improve, standing balance (Lesinski et al. 2015). Furthermore, the present subjects had good balance scores at the commencement of training, which may have provided less scope for further change, i.e. a ceiling effect (Orr et al. 2008), or the quiet bipedal standing task may not have been adequate to challenge the postural control system in these minimally impaired individuals (Clifford and Holder-Powell 2010). It will be of interest to observe the adaptive response in individuals with poorer balance and mobility, possibly after using greater exercise intensities, over a longer training period, and with more challenging assessments of static (i.e. standing on foam) or reactive (i.e. surface perturbations) balance.

Reductions in muscle strength and mass are commonly associated with both fall risk (Benichou and Lord 2016) and mortality (Chuang et al. 2014) in older adults, therefore identifying exercise modalities that limit or reverse functional decline is a priority in ageing research. In agreement with our hypothesis, a very large increase in lower-limb eccentric muscle strength (~ 40–60%; *d* = 1.80–2.04) was detected after both 6-week eccentric training programmes, data consistent with a previous study employing a longer-duration (11 weeks) eccentric training programme in frail older adults (LaStayo et al. 2003). In addition to the rapid and substantial increase in strength, significant increases in VL thickness (9.8–9.9%; *d* = 1.71–2.54) and important functional architectural characteristics (fascicle length, pennation angle) were observed. These findings are consistent with the functional and architectural adaptations observed in young adults after this type and duration of training (Blazevich et al. 2007; Kay et al. 2018), indicating similar adaptive profiles of muscle tissue in young and older adult populations. A significant correlation was observed between changes in muscle thickness and pennation angle (*r* = 0.54; *P* < 0.01), but not between changes in muscle thickness and fascicle length (*r* = 0.35; *P* = 0.07). However, no significant correlations were observed between the improvements in muscle strength or size and mobility. Possibly of greater interest was that although a small but significant reduction in muscle thickness occurred during the detraining period, no significant reduction in strength occurred, indicative that neuromuscular adaptations (Cadore et al. 2014) were important for both the improvement, and then retention, of strength. Regardless, strength (~ 71–85% of the mean increase maintained) and muscle thickness (~ 62–71% of the mean increase preserved) remained significantly greater than baseline after the 8-week detraining period. These are the first data reporting the detraining regression profiles in older adults after a period of isokinetic eccentric training and are indicative of prolonged functional benefit. As limited regression in strength and muscle mass has been reported in younger adults following isotonic eccentric, but not concentric, training (Coratella and Schena 2016), the collective findings of the present study, and those of previous studies, are indicative that the substantial improvements in muscle mass and strength triggered by eccentric exercise are well maintained, with minimal regression after training cessation; this is important information relating to exercise prescription in older adults.

Older adults often present with a fear of falling, poor mobility, and limited aerobic capacity, reducing exercise tolerance and adherence to traditional forms of exercise (LaStayo et al. 2003) that can contribute to the development of frailty (Cesari et al. 2014). However, substantial improvements in muscle strength, size, and mobility were achieved in the present study despite the subjects reporting a low-to-moderate perceived exertion during the training. Furthermore, subjects in the present study were able to accurately achieve the required contraction intensity (i.e. 50% MVC) in > 90% of contractions and had an 89% programme completion rate, with those completing the programme recording 100% adherence to the training sessions. These data are indicative of a well-accepted and tolerated exercise modality, and are consistent with previous studies observing that eccentric exercise was well tolerated in older adults and other individuals with cardiorespiratory impairments (LaStayo et al. 2003; Rooyackers et al. 2003). The improved exercise tolerance is likely a consequence of eccentric contractions having a low metabolic demand, ~ 25% that of concentric exercise of a comparable workload (LaStayo et al. 1999), with the low demand reducing perceived effort and improving tolerance (LaStayo et al. 2003). Similarly, the seated exercise modality used in the present study also eliminated issues with poor mobility and fear of falling, which are common in older populations (LaStayo et al. 2003). Collectively, these findings are indicative that seated isokinetic eccentric exercise mitigates several limitations common to other exercise modes (i.e. aerobic exercise, traditional concentric–eccentric resistance exercise) that compromise older adults’ ability to exercise at a sufficient intensity to provide meaningful adaptations.

In summary, this is the first empirical study to quantitatively evaluate the effects of seated isokinetic eccentric resistance training and, importantly, a period of detraining, on neuromuscular function and musculoskeletal characteristics in older adults. While there were no changes in postural sway metrics following training, a significant reduction in TUG time and substantial increases in strength and muscle thickness occurred after 6 weeks of eccentric resistance training. Notably, these improvements remained significantly elevated after 8 weeks of detraining, indicating a prolonged beneficial effect with limited structural and no functional regression. Importantly, seated exercise removes fear of falling during the exercise and the eccentric modality results in lower cardiovascular demand, enabling subjects to exercise at a low RPE, important for improving exercise tolerance in both healthy and clinical populations. Collectively, these findings have important clinical and practical implications for exercise prescription in older adults that may be prone to neuromuscular and musculoskeletal decline and periods of inactivity, as the functional outcomes as well the exercise modality itself are well suited to the specific exercise needs and physical challenges of older adults. A limitation of the present study is that physical activity was not measured during the detraining period. The minimal regression observed during the detraining period may be a consequence of increased physical activity, maintaining the improvements in functional outcomes rather than a prolonged effect of eccentric training. Future research should compare activity profiles before training and during detraining periods to clarify the potential prolonged impact of eccentric training. A practical limitation of the study is that isokinetic dynamometry equipment is expensive and usually restricted to research or clinical facilities, limiting access for older adults. Future studies should identify alternative strategies to impose eccentric loading to determine the feasibility, acceptability, tolerance, adherence and efficacy in older adults to improve practicality, clinical application and outcomes for older adults.

## Abbreviations

ANOVA: Analysis of variance
COP: Centre of pressure
COP_AP_: Anteroposterior centre of pressure
COP_ML_: Mediolateral centre of pressure
CV: Coefficients of variation
*d*: Cohen’s d
EC: Eyes closed
ECC: Eccentric leg press training programme
ECC_PF_: Eccentric leg press and plantarflexor training programme
EO: Eyes open
FL: Fascicle length
ICC: Intraclass correlation coefficients
MT: Muscle thickness
MVC: Maximal voluntary contraction
*η*_*p*_^2^: Partial eta squared
PA: Pennation angle
RPE: Rate of perceived exertion
SD: Standard deviation
TUG: Timed-up-and-go
VL: Vastus lateralis
